# Digitalizing Health Services by Implementing a Personal Electronic Health Record in Germany: Qualitative Analysis of Fundamental Prerequisites From the Perspective of Selected Experts

**DOI:** 10.2196/15102

**Published:** 2020-01-29

**Authors:** Sabrina Pohlmann, Aline Kunz, Dominik Ose, Eva C Winkler, Antje Brandner, Regina Poss-Doering, Joachim Szecsenyi, Michel Wensing

**Affiliations:** 1 Department of General Practice and Health Services Research University Hospital Heidelberg Heidelberg Germany; 2 Department of Family and Preventive Medicine University of Utah Salt Lake City, UT United States; 3 Department of Medical Oncology National Center for Tumor Diseases, Research Program for Ethics and Patient-Oriented Care University Hospital Heidelberg Heidelberg Germany; 4 Department of Information Technology and Medical Engineering University Hospital Heidelberg Heidelberg Germany

**Keywords:** personal electronic health record, implementation, qualitative analysis, digitalization, health services

## Abstract

**Background:**

The implementation of a personal electronic health record (PHR) is a central objective of digitalization policies in the German health care system. Corresponding legislation was passed with the 2015 Act for Secure Digital Communication and Applications in the Health Sector (eHealth Act). However, compared with other European countries, Germany still lags behind concerning the implementation of a PHR.

**Objective:**

In order to explore potential barriers and facilitators for the adoption of a PHR in routine health care in Germany, this paper aims to identify policies, structures, and practices of the German health care system that influence the uptake and use of a PHR.

**Methods:**

A total of 33 semistructured interviews were conducted with a purposive sample of experts: 23 interviews with different health care professionals and 10 interviews with key actors of the German health care system who were telematics, eHealth, and information technology experts (eHealth experts). The interviews were transcribed verbatim and subjected to a content analysis.

**Results:**

From the expert perspective, a PHR was basically considered desirable and unavoidable. At the same time, a number of challenges for implementation in Germany have been outlined. Three crucial themes emerged: (1) documentation standards: prevailing processes of the analog bureaucratic paper world, (2) interoperability: the plurality of actors and electronic systems, and (3) political structure: the lack of clear political regulations and political incentive structures.

**Conclusions:**

With regard to the implementation of a PHR, an important precondition of a successful digitalization will be the precedent reform of the system to be digitized. Whether the recently passed Act for Faster Appointments and Better Care will be a step in the right direction remains to be seen.

## Introduction

Based on many examples in Europe and beyond, the German government has been pursuing the goal to continue to expand the telematics infrastructure and the introduction of a personal electronic health record (PHR) for all insured persons for several years now [1-3]. Since 2004 and the roll out of the Statutory Health Insurance Modernization Act, the PHR has been regarded as a central feature of the envisaged telematics infrastructure [4]. Since then, starting dates for the implementation of a PHR have been postponed repeatedly.

In the literature, positive developments in European neighboring countries are highlighted as well [5]. Here the understanding of electronic health records is not always clear, both nationally and internationally. Terms and acronyms are often used synonymously and are not always clearly defined. However, meanings often differ in terms of stored data, functions, administration and access rights [6]. A study by the Muench Foundation [2] shows which countries are a nose ahead in the implementation of an electronic health record, which also includes the (patient-administered) PHR. In 2016 and in a follow-up in 2018, a study was carried out in 20 selected European countries to ascertain the status of the introduction of a (personal) electronic health record [2]. On the “European Scorecard,” Germany was shown ranking in the lower midfield. In 2018, Germany almost slipped into the bottom third of the countries surveyed. On the other hand, the Scandinavian countries are the top scorers [2]. While in other countries PHRs have obviously already been established as a central component of national eHealth strategies, in Germany the implementation and application seem to be confronted with certain hurdles. The PHR is regarded as the “supreme discipline” [7] of the digitalization of the German health care system. A recent study analyzed conditions for its success, pointing to central barriers for digitalization in health care [8]. Hesitance with the implementation of a PHR in Germany has been attributed to political framework conditions as a missing or incomplete prerequisite [2,4]. At the same time, the need to relate the implementation more strongly to practical everyday needs and questions of the respective users is propagated [3].

The aim of this study was to explore potential barriers and facilitators for the adoption of a PHR in routine health care in Germany. The PHR here was specified as a Web-based personal electronic health record prototype (Persönliche, einrichtungsübergreifende, elektronische Patientenakte [PEPA], also called a personal electronic health record [PEHR]), where the patient is the owner of their health record [9,10]. This paper focuses on reasons for the failure to implement a PHR in Germany and ventures some ideas to overcome identified obstacles. Therefore, it explores relevant policies, structures, and practices of the German health care system that influence the uptake and use of a PHR.

## Methods

This study was based on semistructured and semistandardized interviews with potential users and eHealth experts. Reporting of this study follows the recommendations of the consolidated criteria for reporting qualitative research checklist (COREQ) [11].

### Study Design

The study is part of the Information Technology for Patient-Oriented Health Care in the Rhine-Neckar Region project, which was funded by the German Federal Ministry of Education and Research (2012-2017) and focused on the structural prerequisites for integrated and cross-sector care of chronically ill people and the scale-up of the prerequisites. A central component of this project was the development of a PHR for use in cancer patients [9,12,13]. Two key features of the PHR are that it is Web-based and patient-administered. Patient-administered means that the patient decides who has access to which parts of the recorded information [12]. Central to the development and application of the PHR is a strong focus on the needs and preferences of end users [9,14]. Based on results from implementation research [15,16], the main objective of the study was to realize a strong user centering and thus include the specific needs and requirements of all PHR user groups in an iterative process. In addition, both the action and work contexts of the user groups as well as relevant structural prerequisites were included in the analysis.

This article focuses on the findings of a subproject in which a usability and feasibility study was conducted. Here the prototype of the PHR was tested and refined with various eHealth experts, health care professionals, and cancer patients involved. In addition to the technical usability, relevant policies, structures, and practices for a successful implementation of a PHR were evaluated [9].

Ethical approval was given by the Ethics Committee of the University of Heidelberg (S-462-2015). All participants gave their written informed consent. The participants’ anonymity and confidentiality were ensured throughout the study.

### Study Sample

#### E-Health Experts

Participants had to be central stakeholders of the German health system or experts in the fields of information technology, telematics, and eHealth. These included organizations like political institutions, federal associations, chambers of commerce, and hospital management. The sampling of participating experts was based on (1) their thematic interest, (2) position or reputation of the specific expert, and (3) potential impact to foster political decisions for a broader PHR implementation. A total of 14 eHealth experts from 14 different institutions were contacted; 3 experts decided not to participate. One expert could not participate in the study for health reasons on short notice. In 4 cases, the contacted persons could name another person in their institution who instead took part in the study. The eHealth experts were recruited by the Department of General Practice and Health Services Research (University Hospital Heidelberg) via postal mail.

#### Health Care Professionals

Based on an intersectoral care setting for cancer patients with colorectal carcinoma developed in this project context, central health care professional groups could be identified and recruited. The study included hospital physicians, nutritionists, and social service workers of the National Center for Tumor Diseases (NCT) in Heidelberg as well as general practitioners (GP) and nonphysician health professionals who work together in the care context of patients with colorectal cancer [9]. All interviewees were recruited using a purposive sampling method. HCPs from the Rhine-Neckar area were contacted according to the research interests. They were identified and selected as stakeholders of PHRs from our research practices and our cooperation partner (NCT). HCPs were recruited by the Department of General Practice and Health Services Research (University Hospital Heidelberg). They were approached face to face or by email.

### Data Collection

The semistandardized and semistructured interviews were conducted in two survey periods. Interviews with the HCPs took place from September 2015 to March 2016. Interviews with the eHealth experts take place between June and October 2016. Interview duration was 30 to 60 minutes.

Prior to the interviews, the HCPs participated in a usability test with the developed PEPA prototype that lasted an average of 35 minutes. Here the HCPs could gather tangible experiences in handling PHRs and formulate idea on positive as well as negative aspects, both in general and specific to their own work context. The focus here was on technical criteria directly related to the manageability of the PHR. The following interviews were expected to generate statements about prerequisites and hurdles beyond pure usability. The usability testing facilitated alignment of barriers and requirements with PHR. The interviews were based on a semistandardized and semistructured pilot-tested interview guide that focused on the main areas of prerequisites and challenges. Themes and questions in this interview guide were based on theoretical considerations and findings from a literature review [17]. The interviews had a duration of 30 to 45 minutes. All but one was conducted in the respective work environment.

Since the experts did not participate in a usability test, a short description of the PHR and access to the PHR internet portals (professional portal for HCPs; patient portal for patients) were sent to the experts prior to the interview. Thus, an overview of the functionality and structure of the PEPA as well as the objectives of the PEPA concept were provided to all interview partners in advance. If requested, a question catalog was sent to the experts beforehand.

The interviews were generally conducted face to face by the first author (SP) at the interviewees’ workplace. Two interviews were conducted by telephone. The interviews lasted 45 to 60 minutes. All interviews were audiotaped and transcribed verbatim but not returned to the participants. The interviews lasted until the saturation of theoretical arguments was reached.

### Data Analysis

The performed qualitative content analysis explored pseudonymized and purged data taken from the original texts [18-20]. A preliminary category system was applied based on theoretical considerations. The category system was adapted during the process of analysis if the data showed additional and new information that required a new category. Therefore, analysis included inductive development as well as deductive application of categories.

In the first step, two interview transcripts from each participant group (HCPs and eHealth experts) were chosen and independently reviewed by the first author (SP), a coauthor (AK), and two other scientific colleagues who were not involved in the project but experienced in qualitative evaluation methods. In the second step, further central topics could be developed, summarized, and labeled based on the temporary category system. The resulting defined codes were differentiated into main and subcategories. Relevant original statements in the transcripts were assigned to these categories. Referencing the developed category system, attributions were repeatedly adapted and condensed in a 3-step discussion procedure with a coauthor (MW). Qualitative content analysis of the data was performed using the software MAXQDA Analytics Pro 18 (VERBI GmbH).

## Results

### Sample Description and General Picture

The study included a total of 10 eHealth experts and 23 HCPs. The eHealth experts were key players in the German health care system working in information technology and telematics at the levels of federal politics, self-administration (federal/state—in Germany, the state sets the legal framework and assignments and the insured people, contributors, and HCPs organize themselves into associations that assume the medical care of the German population on their own responsibility), other associations at the federal level, and hospital management. The HCPs comprised three groups: general practitioners (6), hospital physicians (5), and other health care professions (nonphysician health professional, nutritionists, social service workers; 12).

To gain a general picture of the mood regarding PEPA and its fundamental application in Germany, three pivotal questions were asked in the expert interviews. All the eHealth experts found the application of a PHR generally desirable. When asked whether the prerequisites for an implementation of PHR had already been met, 4 interviewees answered with “rather no,” 3 with “partly,” and another 3 with “rather yes.” This indicates that most of the eHealth experts did not yet or only partially see the most important prerequisite for a successful PHR implementation. When asked whether a PHR could be realistically assessed as a regulatory instrument in 5 to 10 years, almost all respondents answered yes. HCPs also showed a very high acceptance of PEPA. All of the hospital physicians and most of GPs stated that they would use a PHR on a regular basis.

Central prerequisites and associated challenges could be categorized into 3 main topics: (1) documentation standards: prevailing processes of the analog bureaucratic paper world, (2) interoperability: the plurality of actors and electronic systems, and (3) political structure: the lack of clear regulations and incentive structures.

### Documentation Standards: Prevailing Processes of the Analog Bureaucratic Paper World

For most eHealth experts, existing mindsets are still not well matched with digitalization processes. Established procedures of the analog paper world would be prevailing, and electronic duplicates of bureaucratic forms of health care services were leading into the wrong direction. For them it went without saying that the electronic world cannot and should not simply duplicate the paper world of the bureaucracy. If just mirroring bureaucratic forms, the implementation is doomed to failure.

...this can’t work like that. We have a highly structured system that is often perceived as much too bureaucratic, based on the paper world ...all the template forms that exist in [statutory health insurance]–accredited medical care, they all come from the template form commission. They have been decided on, and every field is being discussed...it is impossible and I do not think it makes any sense.... So, you don’t do justice to the patients or their data....

E8

...currently there is a template, don’t know what it is called, 37b or something like that, in triple or quadruple copy, green, blue, pink, or something else... it’s not about mapping this process by a digital document in an electronic health record, but it’s about digitizing the process.

E17

For the eHealth experts, this approach to digitalization was considered wrong and one of the biggest mistakes in the introduction of a PHR. For them, digitalization requires a different logic of how to do things, how to establish standard operation procedures. If analog bureaucratic rationality takes the lead, the project is jeopardized in this perspective. So, to make a PHR a success, existing practices in health care have first to be rationalized, simplified, and redesigned. The right approach, according to the experts, would then be to set up digital structures. A template form with four carbon copies simply should not be duplicated electronically. According to the message of the experts, anyone who stays in the paper world mentally will not be able to realize the advantages of the digital world.

The eHealth Act states that the self-governing body has been commissioned to study the Social Security Statutes (SGB V) and based on its name which of the many forms and so on and so forth could be digitally reproduced. This in a digital age! Basically, it would have to be the other way around, meaning the order would have to be, everything is done digitally, unless there are substantial reasons impeding going digital. Well, that’s the first reason it doesn’t work...we do not think digitally. We think within our analog processes and believe that with a health record, we can electronify the analog processes. That is not the purpose of a health record.

E17

This problem in dealing with digitalization also became evident in a more concrete form at the level of health organizations. HCPs also addressed the multitude of different forms that cannot just be mirrored in the electronic world. It was mentioned by HCPs that the specialized, different documentation standards, especially for clinicians, specialists, and GPs, could constitute a serious barrier to general standards of electronic documentation.

So, my GP still has her paper-based records and if I imagine she has to (change) that, oh God, that would be difficult.

HP02

They also have different documentation, different templates [the clinics].

GP01

Also, documentation is often divided into internal documentation not intended for the patient and external formal documentation. To bring this into a comprehensible and accessible version seemed impossible to many.

...that the patient can read this, not every report is suitable for the patient.

GP02

Yes. You have some things you just don’t want the patient to read now, because it’s really something internal, and that will be difficult, of course.

HP02

In principle, almost everyone was concerned that they would have to deal with a double structure and thus a double workload. Especially since the systems used are tailored to these technical differences and support the differently standardized documentation.

...so, such a double structure that would result, for example, inevitably, that makes double work, so that throws the baby out with the bath water then.

GP03

In this context, the problem of proprietary systems, which are usually incompatible, was often mentioned as well. In almost all cases, physicians agreed to an electronic health record, but when more precise inquiries were made, very often restrictions were mentioned regarding the misleading approach of digitalization. Again, the paper world was not to be duplicated, but a reform of the system deemed necessary to digitize it.

### Interoperability: Plurality of Actors and Electronic Systems

Most eHealth experts agreed that it is a prerequisite for successful implementation of a PHR to reach standards of interoperability. But because of the plurality of electronic systems and actors, including the self-governing bodies, important steps in that direction have not been undertaken so far. Instead of addressing the challenge of interoperability in an accurate manner, isolated solutions were said to be prevailing in Germany. That was mentioned by interviewees when they addressed the status quo of electronic health record applications. It was stated that there were many isolated solutions in Germany including regional projects. The interviewees regretted that it was still impossible to draw these projects onto a supraregional level. The interviewees also criticized the fact that physicians do not have access to the health telematics system. In this context, most of the interviewees emphasized that interoperability must be a first step toward introducing health telematics.

...but if the doctors do not have access to health telematics, then one cannot introduce a health record. The health records are not interoperable, neither technically nor semantically interoperable, and we have a huge problem with that.

E10

The majority of the eHealth experts addressed interoperability as a relevant aspect for the introduction and implementation of a PHR. Basically, almost all interview partners said that there were a multitude of electronic file systems, but in the end interoperability did not exist. This pointed to proprietary business models seen as representative of a fundamental problem for the implementation of a PHR.

...and this also has to do with these proprietary solutions, of course, because the more proprietary systems you have to begin with, the more problems you have as a user. And it doesn’t go back to the fact that the IT technically can’t be solved. But it goes back to the fact that old business models are still very strong and very present there, and in the end prevent a good workflow from developing there. And that is simply something, in my opinion, one cannot somehow preach to the physicians from morning to evening that these IT systems are the future, if they experience in their practice every day that this IT causes problems and doesn’t solve any at all.

E16

As the first developments of digitalization in health services were focused on billing purposes and the digital storage of billing data and processes in general, care and digital processes relevant to care were not considered.

So, one simply does not have the focus on structured filing of medical documents but one has the focus on uniform evaluation standard numbers (EBM-Ziffern)...and if the systems are knitted in this way, then they cannot quickly communicate interoperably with a third-party system....

E8

Most of the HCP interviewees agreed that there are severe problems with integrating different electronic systems. It is said to be a mix of paper and pencil procedures and electronic file systems. Before implementation of a PHR could take place, the uncontrolled growth of different standard operation procedures in the file systems would have to be terminated.

...so I think that...the individual clinic systems or practice systems should be better coordinated with each other.... And, yes, I don’t know how far away the individual providers are from participating, everyone prefers to market their product....

HP10

### Political Structure: Lack of Clear Regulations and Incentive Structures

#### Political Regulations

Many of the eHealth experts and HCPs were arguing in favor of a strong central solution for the problems. Only the legislature would be responsible for providing interoperability. In their mindsets, it was necessary to create legislation and give a clear mandate on who would realize implementation and take responsibility in this context.

...from my point of view, the decisive actor is the Federal Ministry of Health.

E12

I believe that interoperability can only come directly from the legislature. The self-governing body simply has no mandate for this, not derived from the law. In my opinion, the legislature has very hesitantly included interoperability in the eHealth Act. However, this is limited to an archiving interface for patient data-bearing systems. This is a start but is of no use with regard to the health record. The legislature would have to prescribe this directly and then give the order...so to speak provided with this mandate, then specifications gladly can be made together with the industry also....

E8

In addition to interoperability as a central prerequisite for the digitalization of care in general and implementation of a PHR in particular, the clear political will, corresponding laws, procedures, and incentive structures were emphasized. Top-down solutions were generally preferred and seen as harmful only by a minority of eHealth experts. A large number of respondents see that the demand for PHR should originate from patients. It is then the task of politicians, and almost all experts agree on this, to moderate this process and create the necessary framework conditions for it.

I need data protection regulations, I need all the rights and obligations of physicians and service providers, I need a reasonable remuneration system for this, and, in particular, I need the corresponding processes within the health care system behind it: I do that now, and I say:...I’ll push it through and I’ll put it into practice. So, anyone who wants to join is cordially invited to do so, anyone who doesn’t want to join will be left out... Constructive suggestions are welcome; destructive suggestions lead to exclusion. That’s it. And that [the source of these processes] can only be the legislature....

E17

...the patients have to be involved decisively; ...the patient has to play a decisive role.

E16

Almost all interviewees considered the political will on a societal level imperative for the acceptance of a PHR on a broad social basis. Here, the focus was on greater transparency with regard to the discussed contents and decisions. At the same time, citizens should be directly involved in the decision-making processes and the content discussed.

...the question [is], how transparent are the decision-making procedures...that this takes place in a quiet chamber, that the discussion processes are not made public, that is quite unpleasant.... So, I believe that it is much more important to get a higher transparency of the negotiations....

E16

According to the interviewees, the central and first task of the state is to ensure that the patient’s fundamental right to determine disclosure and use of their personal data is respected and legally strengthened. More than half of the eHealth experts considered this aspect to be a central prerequisite for the design and implementation of a PHR.

Well, those are the big challenges, but I think this right to determine disclosure and use of personal data, that’s right at the top....

E6

Basically, I would say that in Germany you sometimes protect the citizen too much from themselves. In this case, I consider the right of informational self-determination to be of more valuable and would in any case plead for it....

E10

At the same time, this was also central to the question of who owns the data. It was propagated that the ownership rights of the data—personal information about and from the patients—must be clarified and defined.

Well, first of all the prerequisite [for] the application of an electronic personal health record is, in my opinion...the patient’s right to data sovereignty.

E17

A similar picture emerges with the HCPs. Many of them also argue that legislatures need to create clear guidelines and responsibilities, especially when it comes to the question of the ownership rights to the data.

Well, the Federal Ministry of Health [certainly] would have to put it on the agenda: “We want that.” Otherwise it will be difficult.

GP08

One thing I have learned in my life: without legislation nothing works at all. Thus, the legislature is challenged. ...[Also] in the context of all these discussions about patient autonomy.... The framework conditions must be created by the legislature. That the patient data...is usually not only in the physician’s practice, but with the patient....

GP01

For most experts, the introduction of a PHR was based on a clear, binding political decision with the allocation and definition of clear responsibilities. Almost all of them pointed to the problem of the normal design and procedures of the health care system in Germany. Specifically, the negotiation habits and procedures of self-administration were addressed.

#### Incentive Structures

With regard to the strongly pronounced sectoral separation in Germany, existing incentive systems within the current system and, above all, false incentives for the introduction of electronic health records were highlighted as relevant obstacles.

A clear specification of the Minister of Health...: “Until then it must come, otherwise there are financial deductions.” Or one could work with a bonus-malus [principle]... Probably this is the only language that hospitals...[and also the outpatient sector understand]. ...One declares: “Until then it must be implemented,” period.

E01

The difficulty of placing this issue in the hands of self-government was pointed out, since in many respects the usual negotiation routines were considered to make the actual process more difficult. One interviewee described this as a “playground” for the “usual games and trench wars.”

The cake must be distributed...these digitalization processes are such a vehicle. So, it’s a new playground that’s being used for that.

E6

In order to keep PHR systems running, carrot and stick policies on the provider’s side were contemplated as well. Interviewees mentioned the various remuneration systems and incentive structures that in their opinion would change the mindset of those HCPs who do not support the implementation of a PHR.

I have come to the point where I believe that certain processes, certain digitalization processes cannot be left to the regular structures of our health system. ...This is such a profound infrastructural change that I believe it must be pushed through relatively hard by the state with a carrot and stick system.

E6

One interviewee described the regulation of performance reimbursement as “the manifestation of isolation.” Due to the different performance reimbursements, conduct of the individual HCP was seen as being structured in a way that imposes a severe obstacle for the implementation of the PHR. Different fees thus would determine to what extent and in what way communication between HCPs is desired and in what direction it would take place.

Well, when the data flows, there’s always the danger...that the money flows afterwards. That is what the sectors are striving for. ...And—who has the data, has the power.

E6

In this context, the sender-receiver problem was addressed, in which the sender is usually the disadvantaged person, while the receiver attains the greater benefit from digitalization, which must be compensated for. This means that one part of the medical profession feels more disadvantaged than another.

We often have an imbalance in benefits, so that we have to somehow create incentives or a balance in order to start the whole thing in the first place. ...Especially [among] GPs and specialists, because some always see themselves as transmitters and others as receivers. Then you get into a situation where the physicians, who were originally able to work well together, first see themselves as opposing actors. Because you simply haven’t created the setting that gives them the feeling, to take this digitalization step equally.

E8

Not only will a clear position of the politicians on this topic be expected, but also clear guidelines up to the presentation of a model process (eg, for hospitals).

So, one [the legislature] should prestructure the whole process unanimously also times as a pattern [process].

E1

## Discussion

### Principal Findings

The study aimed to explore potential barriers and facilitators for the adoption of a PHR in routine health care in Germany. This paper focuses on reasons for the failure to implement a PHR in Germany and ventures some ideas to overcome identified obstacles. Therefore, it explores relevant policies, structures, and practices of the German health care system that influence the uptake and use of a PHR.

The results showed that organizational, economic, and political interrelations become relevant. Three themes emerged to be central to the implementation of a PHR in Germany: (1) documentation standards: prevailing processes of the analog bureaucratic paper world, (2) interoperability: the plurality of actors and electronic systems, and (3) political structure: the lack of clear regulations and incentive structures (Textbox 1).

**Principal findings: categories and themes.**

Documentation standards:

- Highly segmented health care
- Mandate of the analog paper world
- Highly bureaucratic
- Strongly differentiated
- Various standards and terminologies

Interoperability:

- Federal structures
- Highly segmented health care
- Plurality of electronic systems
- Old business models
- Plurality of actors
- Semantic and technical interoperability

Political structure:

- Clear political will
- Incentive structure
- Rights and regulations
- Self-administration
- Different interests
- Strong advocacy groups
- Clear responsibilities
- Transparency

#### Documentation Standards: Prevailing Processes of the Analog Bureaucratic Paper World

Starting from a highly segmented care landscape, the various standards in medical documentation represent a central challenge for the implementation of a cross-sectoral application of a PHR. The differentiated use of documentation can therefore often be attributed to the differentiation between inpatient and outpatient care, as well as to the various specializations. The result is a multitude of distinguished forms and heterogeneous terminologies. In literature, this is often discussed in the context of semantic interoperability, which is considered a prerequisite to be able to guarantee the targeted exchange of patient-related documentation at all [21,22]. The highly bureaucratic procedures in dealing with documentation make the transformation of the analog paper world into the electronic form of a PHR considerably more difficult. If patients are included in the exchange of information, these challenges were specified in terms of health literacy [23].

It will be important to take these prerequisites into account when designing the digitalization of the analog paper world. The pure duplication of the most varied documentation standards and approaches from the paper world continues to lead to challenges for the creation of semantic interoperability [21,22]. Rather, it becomes necessary to adopt digital processes. As literature in the context of digital medicine has shown, a common technical language as well as concrete guidelines for the exchange formats of this data will be inevitable for a successful adoption of digital technologies [24]. A reform and reorganization of previous standards and processes as well as a different understanding of digitalization will be unavoidable. Different administrative practice and clinic systems only reproduce the variety and diversity of existing standards. The challenges already mentioned for the documentation behavior of physicians with regard to the use of a PHR [25-27] are partially confirmed by our results (eg, in the case of documentation quality, double structure/double documentation standards, misinterpretations). The current market situation for electronic health records reflects this diversity of existing standards and the difficulty of linking them.

#### Interoperability: Plurality of Actors and Electronic Systems

In the context of a segmented and federal health care landscape with a variety of different documentation systems and a multitude of providers, interoperability is a major challenge. Interoperability can be understood as the ability of independent, heterogeneous systems to work together as seamlessly as possible in order to exchange information in an efficient and usable manner or to make it available to the user [21]. Statement papers in the political discussion generally propagate the development of interoperability as a central condition for the application of a PHR: “We want to create new approval paths for digital applications, establish interoperability, and strengthen digital security in the health care system” [1]. Interoperability stands for overcoming the often portrayed island solutions, which have so far been predominant in Germany [4].

The existence of a multitude of electronic systems and proprietary business models makes it difficult to implement interoperable systems. Uniform technical standards and interfaces for the exchange of data cannot yet be guaranteed in the way necessary for the use of a PHR. In order to enable digital structures and practices, as it is necessary in the case of PHR, the literature speaks, among other things, of creating uniform industry standards to enable interoperable systems [2,8].

However, as the findings of this study show, it seems not to be enough to recognize and solve interoperability as a purely technical challenge. Semantic and technical interoperability must therefore go hand in hand [21], which entails a far-reaching reorganization of the bureaucratic paper world and the associated restructuring of structures and practices in health care. Clarifying the market situation by creating a telematics infrastructure will be as important as standardizing and simplifying documentation methods.

#### Political Structure: Lack of Clear Political Regulations and Political Incentive Structures

The typical self-administration structure of the German health care system enables strong stakeholder participation in decision-making processes [28]. Similarly, as in the case of a PHR, there is a risk that negotiation routines and diverging interests will hamper a clear line in the implementation and design of a PHR. As propagated from the experts, a greater legislative responsibility could speed up the implementation process and ensure a consistent and clear approach. Also, in literature, a uniform policy strategy with clear responsibilities as well as a greater transparency for citizens and their involvement is needed for a sustainable digitalization of health care and implementation of a PHR [8]. From an expert’s point of view, it is necessary to develop an overall strategy based on the eHealth Act, particularly in a complex self-administration structure like in Germany. This also includes the formulation and allocation of clear responsibilities with clearly framed tasks and objectives [2,8]. A relationship management [8] that bundles and moderates the various interests, goals, and opportunities is equally proposed. Some literature provides incentives for the use of a PHR for the respective user groups [8]. As a rule, the focus here is on monetary incentives, which should be available for doctors, as well as sanctions, if the expectations of use cannot be fulfilled [8]. The experts mentioned that necessary negotiations between relevant stakeholders of the health care system should include the persisting differences into existing incentives. Compensatory incentive and sanction structures should be created to enable potential user groups to benefit from PHR on a lending basis.

Confirming findings of international studies in which data protection raised only moderate concerns among potential user groups [6,24-26], data protection does not play a significant role for the interviewed experts in this study either. Even though data protection was described as too outdated and not dynamic enough by some voices, especially with regard to international standards, due to the German special way it does not represent a major challenge for the implementation of the PHR. In fact, experts believed that data protection is often misused as an argument in connection with digitalization in the health care sector in order to prevent things from happening or to avoid having to discuss relevant and more difficult issues.

### Strengths and Limitations

Besides the early and consistent involvement of end users and their action and working contexts in the development of the PHR, it was another strength of this study to include the general policies, structures, and practices of the German health care system in the evaluation. Thus, central conditions of the German health care system can be examined at different levels, and their relevance for the implementation of PHR can be contemplated.

As explained above, the understanding of a PHR varies both internationally and nationally. Although before the interviews with the experts there were efforts to present the PHR concept defined in the project context in written form, the arguments of the experts can also refer to other concepts of a PHR. Even though fundamental questions on the PHR concept were asked or answered before the interviews, it cannot be conclusively ensured that the answers relate exclusively to this concept.

The fact that all HCPs included in the study work in the field of colorectal cancer may restrict the generalizability and transferability of the results to other (medical) settings.

### Conclusions

For the eHealth experts in Germany, a PHR is basically desirable and unavoidable. At the same time, a number of challenges for implementation in Germany have been outlined which can be taken into future focus.

Whether the recently adopted Act for Faster Appointments and Better Care (appointment and care law, TSVG), which came into force on May 11, 2019, is a step in the right direction remains to be seen. It obliges all health insurance companies to offer electronic patient records for their insured by 2021 at the latest. In addition, the legislature assumes primary responsibility related to the creation of defined interfaces to enable interoperability. It will depend, among other things, on how the market situation clarifies itself and how patient autonomy can be strengthened under the given conditions.

In principle, it will be important to consider existing structures of the medical care landscape and the effects of digitalization processes on these structures when introducing the PHR. Especially with regard to the implementation of a PHR, one important precondition of a successful digitalization will be the precedent reform of the system to be digitized.

## Abbreviations

COREQ: consolidated criteria for reporting qualitative research checklist
GP: general practitioner
HCP: health care professional
NCT: National Center for Tumor Diseases
PEHR: personal electronic health record
PEPA: Persönliche, einrichtungsübergreifende, elektronische Patientenakte
PHR: personal electronic health record
